# Student perceptions of STEM engagement and student-led learning in a biotechnology-focused honors course: a qualitative case study

**DOI:** 10.3389/feduc.2026.1777676

**Published:** 2026-06-30

**Authors:** Molly E. Tuck, Kaylee A. Palomino, Luke H. Bradley

**Affiliations:** 1 University of Kentucky START Program, Lexington, KY, United States,; 2 Lewis Honors College, University of Kentucky, Lexington, KY, United States,; 3 Department of Neuroscience, University of Kentucky College of Medicine, Lexington, KY, United States

**Keywords:** authentic learning, honors college, interdisciplinary, student-led inquiry, transdisciplinary education

## Abstract

“Honors 152: What is possible? Developments in Biotechnology” is a course tailored for introductory students of all majors to promote self-guided exploration of science, technology, engineering and mathematics (STEM). By introducing academic journal articles early in the semester and using authentic learning strategies, the course helps students grow more confident in exploring unfamiliar topics in biotechnology. Students demonstrate their learning through team presentations, mid-term examinations, and a final project, which involves independent research and presentations on chosen biotechnology topics. Rather than following detailed assignment requirements, the course scaffolds peer learning and student-selected inquiry to reduce perceived stress and encourage students to engage with unfamiliar biotechnology topics. To examine former students’ perceptions of the course, we conducted a qualitative case study using open-ended survey responses from students across three recent cohorts. Responses highlighted the value of peer learning, increased comfort with complex or unfamiliar STEM topics, and lowered perceived stress relative to traditional course structures. In addition, several respondents also described a greater openness to STEM with a greater interest in pursuing undergraduate research or STEM coursework. These findings suggest that early Honors courses can serve as a model for an early-stage Honors intervention to provide meaningful opportunities for students to practice autonomy, inquiry, and scientific communication, while also identifying course design features that may be useful for other Honors STEM educators.

## Introduction

Honors programs are often viewed as high-impact environments for building undergraduate inquiry, academic identity, and long-term engagement, all benefits that can extend well beyond graduation (Miller et al., 2021a; Miller and Dumford, 2018; Rinn and Plucker, 2019). Prior work suggests that when Honors students engage meaningfully in research, they often report reaffirmed commitment to their majors and an increased sense of purpose (Medaille et al., 2021). Undergraduate research is widely recognized as important for developing research confidence, disciplinary identity, and persistence (Craney et al., 2011; Davis and Wagner, 2019; Palmer et al., 2015, 2018). Yet, research experiences in many Honors pathways are still concentrated near the later stages of their college programs (e.g., theses or capstones), potentially missing an important opportunity when students are first forming their academic identities and deciding whether STEM disciplines feel accessible or relevant. Early engagement is particularly important in STEM, where introductory experiences can influence both attraction and retention (Ellerton et al., 2016) and where belonging and engagement are central to persistence, especially for students traditionally underrepresented in STEM (Bradley et al., 2021; Christe, 2013; Jong et al., 2020; Thiry et al., 2011). In this context, understanding how early Honors courses influence student development, and identifying which course elements students perceive as most valuable, remains an important need in education research (Rinn and Plucker, 2019).

At the course level, this need intersects with longstanding questions about how teaching strategies and curriculum shape engagement in both general and Honors educational settings (Chaney et al., 2021; Glynn et al., 2005; Miller et al., 2021b; Weissman and Boning, 2003). While Honors participation has been linked to outcomes such as collaborative learning, reflective and integrative learning, and student–faculty interaction (Bertrand and Slovensky, 2020; Miller and Dumford, 2018), the best conditions for supporting these outcomes, especially in early STEM-focused courses, are still emerging. This gap is particularly relevant as high-achieving students often experience elevated stress connected to perfectionism and evaluative demands (Cross et al., 2018; Rice et al., 2015), with traditional assessments and examinations amplifying perceived stress for some learners (Evans et al., 2020). Thus, STEM-focused recommendations increasingly emphasize supportive structures that preserve academic rigor while actively reducing anxiety through connection, group work, and active learning (Hsu and Goldsmith, 2021).

Authentic learning, which provides students with real-world examples and decision-making opportunities, offers an approach for designing early Honors experiences that build student confidence while supporting engagement. In this manuscript, we use “authentic learning” to refer to course activities that ask students to engage in practices resembling those used by scientists and scholars, including reading primary literature, identifying questions, synthesizing evidence, and communicating findings to others. Across higher education, authentic learning has been associated with improved academic performance and learning outcomes, as well as greater retention when students apply knowledge outside the classroom (Zielinski, 2017). Students also gain a stronger sense of self (Roach et al., 2018) and employability-related skills (Ornellas et al., 2019). In STEM, authentic projects can strengthen identity and shape major and career interests while aligning with competencies and goals (Beier et al., 2018; Reynolds and Kearns, 2017; Singer et al., 2020), particularly when tasks are realistic and students feel ownership of their learning (Roach et al., 2018).

Honors 152: *What is possible? Developments in Biotechnology* (HON 152) was therefore designed, not as a substitute for formal undergraduate research experience, but as an early course-based introduction to research-adjacent practices at the intersection of Honors education and authentic learning. Offered through the University of Kentucky’s Lewis Honors College, HON 152 introduces early Honors students across all majors to biotechnology through primary literature, collaborative learning, student-led inquiry, and scientific communication. We define student-led inquiry as a process in which students select, investigate, organize, and communicate a biotechnology topic of their choosing with faculty support, but without a narrowly prescribed topic or format. Students engage with peer-reviewed articles early in the semester in teams, complete a midterm exam that tests their ability to interpret a current research article, and finish with a self-directed final project based on any biotechnology-related topic of their choosing. Projects include both a paper (based on current peer-reviewed journal author guidelines) and an individual presentation. By intentionally minimizing prescriptive project requirements and emphasizing independence and peer learning, the course is designed to build confidence in scientific material, develop research-related skills, and sustained interest in biotechnology, without adding unnecessary performance pressure.

While the benefits of authentic learning in STEM education are well documented, less is known about how introductory Honors courses that emphasize student-directed inquiry shape students’ perceived stress, confidence with scientific material, engagement with research-related practices, and longer-term interest in STEM, especially for students in non-STEM majors. To gain insight, we conducted an exploratory qualitative study of former HON 152 students from three recent cohorts. The study focuses on two central questions: 1: What did students identify as the most impactful aspects of the class? and 2: How did students navigate the self-directed learning components of the final assignments?.

## Methods

### Participants

Participants of this study were students enrolled in the last three offerings of the HON 152 course (Fall 2021, Fall 2022, and Fall 2023) and who opted to participate in the anonymous survey on their experiences and perceptions of the class. Of the 68 students contacted across the three cohorts, 13 students responded to the survey. This represents a response rate of approximately 19%. Because of this response rate, the findings are interpreted as a qualitative case study of respondent perceptions rather than representative evidence of all students who enrolled in HON 152. Approximately 69% (*n* = 9) indicated that they had taken the course in their freshman year while approximately 31% (*n* = 4) had taken the course during their sophomore year. Additionally, 38% (*n* = 5) responded to the survey in the same year they had taken HON 152, and 62% (*n* = 8) of students had taken the course one to two years before participating in the study. These differences in time since course completion may have shaped responses, as some students reflected on the course shortly after completion, while others interpreted the course through subsequent academic and research experiences. Finally, although HON 152 is taught by a STEM professor, it is open to students of all majors. Therefore, students were asked to indicate whether they considered their major to be in the STEM field for the researchers to get a better understanding of whether experiences differed between STEM and non-STEM students. From the sample, 69% (*n* = 9) stated that they were a STEM major while 31% (*n* = 4) were not and, when asked what their majors were, two stated that they were “pre-law” while one was majoring in “communications” and the other was in “business.”

### Data collection

The professor who teaches the HON 152 section sent out an anonymous survey to all students previously enrolled in the last three cohorts of the class in February 2024. The survey consisted of nine open-ended questions regarding their experiences and perceptions of various curricular aspects of the course. These questions were as follows:

1. What was your motivation for taking HON 152 when you originally registered for the course?
2. Prior to the HON 152 course starting, how did you feel about biotechnology?
3. Looking back on the HON 152 class, as a whole, what aspects of the class had the greatest impact on your experience and learning?
4. What were your biggest takeaways from the HON 152 course?
5. For the final assignment of the HON 152 class (which included picking a topic related to biotechnology, presenting on the topic, and writing a paper), how did you choose your topic?
6. Without a rubric for the final HON 152 assignment, how did you determine what you would include in your final project?
7. How would you describe the experience of reporting on your final HON 152 topic, either in the presentation or in the paper?
8. Since taking the HON 152 class (whether it has been a semester or a few years), how have you engaged in conducting research as an undergraduate student, if at all?
9. Have you taken STEM classes since HON 152, and what are your current views on taking STEM classes?

At the end of the survey, space was also provided for students’ additional comments and thoughts that were not applicable to the previous questions. Students were given two weeks to opt into the study by filling out and submitting the survey.

### Qualitative analytic approach

This study used an exploratory qualitative case study design with analysis of open-ended survey responses. The survey questions were intended to generate retrospective reflections on students’ experiences in HON 152. Open-ended questions were selected because they allowed respondents to identify course elements they found meaningful in their own words, rather than requiring them to select from predetermined responses.

Responses were reviewed and coded by the two researchers who did not teach the course to ensure participant anonymity and reduce instructor-related bias. Coding was primarily inductive, with initial codes generated from recurring ideas in student responses. The researchers then compared their interpretations and grouped similar responses into underlying themes aligned with the research questions. Differences in interpretation were resolved through discussion and consensus. Where relevant, responses were also compared descriptively between STEM and non-STEM majors. Because the purpose of the study was exploratory and qualitative rather than a quantitative measurement, reliability statistics were not calculated. To clarify how the course design aligned with the study’s research questions, Table 1 summarizes the major HON 152 course components, intended learning outcomes, and student-led or authentic learning elements.

## Results

### First research question

In examining responses to the first research question, three elements of the course were frequently identified by respondents as being particularly impactful. Because not all respondents answered each item in equal detail, counts are reported where appropriate to indicate how many respondents contributed to each theme. These elemental aspects were learning with and from classmates, the focus of the class being outside of students’ typical comfort zones, and the design of the midterm and final assignments.

#### Peer and collaborative learning

We use this label to refer to students’ reported learning with and from classmates, not to indicate formal implementation of the Team-Based Learning pedagogical model. Five of the student responses focused on the impact of their peers in learning about biotechnology. One student noted, “I think seeing first-hand the potential for human creativity regardless of field was impactful. I saw that the entire class was able to come up with ideas and problems that needed to be addressed just like scientists.” Another student responded that the people in the class, both their peers and the professor of the course, had the greatest impact on their learning.

Additionally, many students specifically pointed to the group work in the classroom, with a respondent terming it as “team-based learning.” One student noted “the group work helped for me to see biotechnology through the lenses of other majors and helped for me to have a better overall view of its applications.” Another student stated that, by having the support of working toward a task with their group, they had the research freedom to “go down ‘rabbit holes’ and research tangentially related topics [in biotechnology].” Finally, two of the respondents stated that working in groups helped with choosing project topics, in the absence of a detailed grading rubric or topic requirements from the professor, which will be discussed later in greater detail.

#### Leaving comfort zones

Many of the students did not indicate having a background in biotechnology prior to enrolling in the class. Because of this, some students noted an initial discomfort with STEM classes, especially for students who were not enrolled in a STEM major when they participated in the HON 152 class. One student, who was majoring in Communications, said “I was familiar with STEM based classes but not comfortable taking them. Those classes were not my favorite.” Similarly, a student who had been enrolled in a pre-law major wrote, “I did not plan on entering STEM during or after college.”

Yet, for many students, the need to move outside of their comfort zone to engage in the section’s theme of biotechnology was noted as a strength of the overall course. One student said they found the class to be very engaging because “we were forced to learn things outside of our comfort zones by doing presentations.” The same Communications major who was initially uncomfortable taking STEM courses noted that the most beneficial aspect of the course was learning, “that I don’t have to know every in and out of a topic. Getting an overall understanding of something in a field that I am not familiar in is still enough to have meaningful conversations and helps my learning.” Another student wrote, “the daily discussions urged students to think outside the box about ways in which biotechnology could be applicable to daily life.” In fact, many students noted that their biggest takeaway from the course was becoming more aware of the presence of biotechnology in their everyday routines. One student applied this experience to how they plan to approach future classes, stating:

“The biggest takeaway I get from HON-152 was that despite how difficult the research that is going on today seems, all of it is based on simple mechanisms that combine to form something more complex. Nothing then becomes too difficult for me to understand, as long as I get the basic mechanisms and ideas.”

Eleven of 13 respondents either reported subsequent participation in undergraduate research or expressed interest in participating. Because this item combines actual participation and interest, we interpret it as evidence of continued openness to research rather than as a measured research-participation outcome. Similarly, of the 11 students who responded to Question 9, 10 reported taking at least one STEM class after HON 152. For example, the two pre-law majors each reported further engagement in STEM. One student wrote, “I think STEM classes are fun and can teach you a lot if you buy into the class,” while the other stated, “I view taking STEM classes as valuable for all individuals: a well-rounded education is key for a good critical thinker and learner.”

#### Design of assignments

Students also commonly referred to the design of the midterm and final assignments when discussing the beneficial elements of the class. Given that these assignments minimized prescriptive rubric criteria and allowed students substantial choice in topic and structure of the presentation or paper, some student respondents described feeling empowered to direct their own learning. One student wrote:

“The individual project towards the end of the semester that challenged each of us to research a topic of our own and create presentations on future application of how we could solve the issue with biotechnology was a great way to expand our thought processes and see what we cared about since the topic was of our own choosing.”

Another student noted that, because there was not a formal rubric, they did not feel pressure from the assignment. A similar sentiment was shared by a different student who noted, “The presentation was not bad and did not stress me out, the class was a very judgement free zone.”

The lack of perceived stress was a reoccurring response to Question 7, regarding their experiences with reporting on the final topic. In total, five students mentioned either feeling no stress or less stress than a typical college course due to the design of the assignment. However, given that the assignment designs broke from conventional college requirements, some students did mention early anxiety from the lack of guidance. One student wrote:

“I thought it was daunting yet liberating how we did not have a strict rubric to follow. It was difficult because I wasn’t sure what I needed to include but it was liberating because I was just able to figure out the most important aspects of my topic and how it related to the class and the real world. I liked having the freedom to choose a topic and really get creative with it.”

Many students noted that the professor also played a role in the success of the assignment design. One student stated that their greatest takeaway was, “how a kind of teacher can make a class something you care about and are excited to learn about.”

### Second research question

In addition to examining students’ general takeaways from the HON 152 course, the researchers also wanted to better understand how students navigated the self-led aspects of the assignments. Based on responses to the survey, we found several themes related to the second research question. First, six students described selecting their project topic based on personal interests, while four of the respondents chose the topic based off of relevance to their major, future goals, or everyday life. Second, many students chose a specific format for their presentation. Finally, some students had conflicting experiences between the paper and presentation formats of the project.

#### Topic interest and relevance

When asked how they came to decide on their project topic, six of the students noted that it was based on their interests. One student described the process as, “I chose my topic by writing a list of medical technology that I was interested in and tried to determine I would be most interested in researching.” Another student stated, “I chose my topic by doing a Google search and finding a topic that I thought was interesting. It didn’t have much to do with my major, but I enjoyed the freedom of getting to learn about something new.”

The next most common response was based upon relevance, with four students noting this approach. However, within the theme of relevance, there was some variance, with some students looking for relevance to their major and others looking for relevance to their lives. In describing relevance to their major, one student responded, “My primary rational while choosing my topic was wanting to combine biotechnology with my actual major: computer science. Data storage using DNA was the only topic I could think of that incorporated both subject areas.” On the other hand, one student stated, “I chose my topic based on something we use in our everyday life and I chose something I did not know much about but something that affected me.”

#### Format matters

To help narrow down some ambiguity with the project, some students chose to base their project off a specific format. One student responded that their goal was to have their essay resemble a product review of their topic, which led to a general outline comprised of three parts: an overview, a comparison of the pros and cons, and major takeaways of researching the topic. Another student chose to format their paper like a research journal entry and stated, “so I got my inspiration from a science journal that I found online. It had an abstract, and different sections about the biotechnology, as well as a conclusion.” Still another student decided to treat their project “like an analysis and comparison of different research papers already published.”

Still, while others did not reference a specific format, several student respondents mentioned starting with an outline to structure their project and help determine what information to include. One student described the process of devising this less formal format as “I thought about subjects related to my topic, such as how my topic was accomplished, relevant industry factors, new technology and studies.” Another student noted, “I included enough information so that the project was understandable and in context. I just included what I though made sense, and left out a lot of the smaller details.” Yet, despite the assignment being designed to de-emphasize grades and promote self-led learning, not all students embraced this approach. One student admitted they “went in depth as possible to let the idea carry us through a good grade.”

#### Preferences between papers and presentations

As the final project consisted of both a paper and a presentation, it became apparent that some students preferred the paper over the presentation or vice versa. Two of the students articulated a preference for the paper format while three commented on favoring the presentation.

The two students that preferred the paper indicated that there was more room for topic exploration and understanding previous scholars’ approaches to the work. One student wrote:

“The presentation was an average experience. I just researched the topic and presented as I had been doing the entire semester. The paper was more interesting because I really had to dig deeper to try and understand the format research papers and review papers take in presenting their work. For that I had to look through multiple papers which was a beneficial experience to have.”

Similarly, another student echoed this as a benefit of the paper, although the difficulties of the presentation were situated to the context of that specific time. They stated:

“The paper was fascinating to write; the process involved cross-referencing different research teams’ approaches to DNA storage, then coming up with a cohesive narrative. The presentation was admittedly less enjoyable. My finals during the [semester] took more time to prepare for than I anticipated, leaving me little time to create both my PowerPoint and practice presenting. The final presentation did not go as smoothly as I’d hoped, but I still enjoyed reporting on the final topic overall.”

Two student respondents stated an obvious preference for the presentation in developing their skills beyond content knowledge. One student specifically identified the need for learning public speaking skills:

“The aspects of presenting and the midterms/finals had the greatest impact on my experience and learning. I believe that public speaking skills are important for all individuals, and it taught me how to design a presentation and present it well on a topic I was somewhat new to.”

Additionally, two students mentioned the presentations when describing areas of the course that produced less perceived stress. One noted, “I would describe the experience of reporting on my final topic as painless. The presentation was not bad and did not stress me out,” while the other stated, “I thought this was a very good way to have a final because the presentation felt very low stakes and it made finals season a lot less stressful.”

## Discussion

Respondents’ reflections suggest that several features of HON 152 may have supported student-led learning, lowered perceived stress, and continued interest in STEM coursework and undergraduate research. As summarized in Table 1, HON 152 combined peer-reviewed article discussions, collaborative learning, analysis of current literature, and a self-selected final project to scaffold student autonomy and scientific communication. Students’ reflections suggest that these design features may have supported peer learning and comfort with unfamiliar STEM material. In both instances, students acknowledged that this reflected the professor, who created a classroom environment that supported student autonomy. These findings align with previous research on authentic learning that emphasizes the importance of collaboration (Cross et al., 2018; Schwortz and Burrows, 2021) and student buy-in (Roach et al., 2018).

Interestingly, the thematic challenges of the course appeared to be an engaging element for students. This finding complicates concerns that challenging STEM content necessarily increases student perceived stress, suggesting that some respondents perceived advanced material as manageable when paired with autonomy, peer support, and a low-stakes classroom environment (Rice et al., 2015). Instead, it appears that coupling advanced content with a supportive environment and an emphasis on learning rather than grades may contribute to a lower perceived stress, encouraging environment for some students to venture outside of the comforts of previously learned material. This finding was evident in student responses on how they chose their project topics, with the majority choosing either something of interest or something with personal relevance, rather than simply selecting topics they knew well and felt confident in delivering. The timing played an important role in the design because these assignments occurred during midterm and at the final stage of the course, this provided the professor adequate time to encourage a judgement-free classroom environment and highlight the connections between the topic and students’ interests.

Furthermore, students’ use of formats in designing their final projects suggests the importance of providing resources when implementing student-led assignments in honors courses. While students appreciated a lack of rubric while engaging in the assignment, many noted the benefits of having formatted resources from which to draw inspiration. However, given the wide range of directions in which students took their projects, instructors should similarly provide diverse types of resources to empower students.

Finally, the findings have implications for disciplinary inclusivity within Honors STEM courses. Because HON 152 enrolls students across majors, the course offers a model for introducing non-STEM students to biotechnology without requiring prior confidence or a commitment to a STEM major. Responses from non-STEM majors, including students in communications, pre-law, and business, suggest that some students perceived value in engaging with STEM topics as part of their broader Honors education. However, because this study did not collect demographic data beyond academic major category, we do not make claims about demographic diversity or differential effects by other identity categories.

### Limitations and future directions

This study has several limitations. First, the findings are not intended to be generalizable to all students who took HON 152 or to all Honors STEM courses. The study used a qualitative case study design to examine respondent perceptions of one course at one institution. Second, the response rate was low: 13 of 68 students contacted completed the survey, yielding a response rate of approximately 19%. This creates the possibility of self-selection bias, as students with specifically positive or negative experiences may have been more likely to respond. Third, the study used convenience sampling because all students from the three most recent cohorts were invited to participate and respondents self-selected into the survey (Stratton, 2021).

Fourth, responses were retrospective. Some students completed the survey during the same academic year in which they took HON 152, while others responded one to two years later. These differences may have affected recall and may have shaped how students connected the course to later coursework, research experiences, or academic interests. Fifth, the survey was not a validated assessment instrument and should not be interpreted as measuring course outcomes quantitatively. Instead, it was used as an exploratory qualitative tool to identify themes in former students’ reflections. Finally, demographic data beyond STEM/non-STEM major status were not collected, limiting our ability to examine how students’ experiences may have differed by race, gender, first-generation status, or other identity categories.

Future research could build on this case study by collecting data across multiple course offerings, increasing response rates through additional recruitment strategies, comparing student reflections before and after the course, and developing a validated instrument to examine whether similar Honors STEM courses achieve their intended learning outcomes and serve as a model for an early-stage Honors intervention. Studies across different institutional contexts, including smaller colleges, non-R1 institutions, and Honors programs with different curricular structures, would also help clarify the transferability of this course model.

## Figures and Tables

**TABLE 1:** HON 152 course components, intended learning outcomes, and student-led/authentic learning elements.

Course component	Intended learning outcome	Student-led/authentic learning element
Early peer-reviewed article discussions	Build confidence reading and discussing scientific literature	Students interpret biotechnology research with peers
Group discussions and presentations	Develop collaborative learning and scientific communication	Students explain biotechnology concepts to classmates
Midterm article analysis	Practice reading, summarizing, and interpreting current research	Students analyze a current peer-reviewed article
Self-selected final project	Support autonomy, inquiry, and topic ownership	Students choose a biotechnology topic of their interest
Final paper and presentation	Communicate scientific ideas in written and oral formats	Students synthesize sources and present findings to peers

## Data Availability

The raw data supporting the conclusions of this article will be made available by the authors, without undue reservation.
